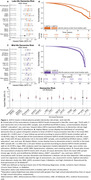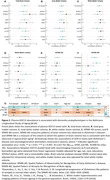# Multi‐cohort analyses link plasma GDF15 with dementia, brain atrophy, and plasma biomarkers

**DOI:** 10.1002/alz.086953

**Published:** 2025-01-09

**Authors:** Cassandra O Blew, Michael R Duggan, Cassandra M Joynes, Gabriela T Gomez, Qu Tian, Luke C Pilling, Jingsha Chen, Mary R Rooney, Pascal Schlosser, Alison B Herman, Dimitrios Tsitsipatis, Myriam Fornage, Rebecca F. Gottesman, Priya Palta, Yifei Lu, Christie M Ballantyne, Michael E Griswold, Josef Coresh, Keenan A. Walker

**Affiliations:** ^1^ Laboratory of Behavioral Neuroscience, National Institute on Aging, Intramural Research Program, Baltimore, MD USA; ^2^ Johns Hopkins University School of Medicine, Baltimore, MD USA; ^3^ National Institute on Aging, Baltimore, MD USA; ^4^ University of Exeter, Exeter, Devon United Kingdom; ^5^ Johns Hopkins Bloomberg School of Public Health, Baltimore, MD USA; ^6^ National Institute on Aging, Intramural Research Program, Baltimore, MD USA; ^7^ University of Texas Health Science Center at Houston, Houston, TX USA; ^8^ National Institute of Neurological Disorders & Stroke Intramural Research Program, National Institute of Health, Bethesda, MD USA; ^9^ University of North Carolina Chapel Hill, Chapel Hill, NC USA; ^10^ Baylor College of Medicine, Houston, TX USA; ^11^ University of Mississippi Medical Center, Jackson, MS USA

## Abstract

**Background:**

Growth/differentiation factor‐15 (GDF15) has been associated with dementia risk, yet its predictive value across cohorts and sub‐population, as well as its relationship with endophenotypes relevant to dementia, remains unknown.

**Methods:**

Using the Atherosclerosis Risk in Communities (ARIC) study as the discovery cohort, we examined the relationship between plasma GDF15 levels (SomaScan) and risk for incident all‐cause dementia (ACD) in late‐life (N=4,287, 7‐year follow‐up, M_age_=75±5) and in midlife (N=11,595, 20‐year follow‐up, M_age_=57±6). Utilizing the UK Biobank (UKB; replication cohort), we related plasma GDF15 (Olink) to incident ACD (N=35,673, 14‐year follow‐up, M_age_=61±5), vascular dementia (VaD) and Alzheimer’s disease dementia (AD). Finally, we examined the cross‐sectional association of plasma GDF15 (SomaScan) with brain volume (N=994), white matter lesions (N=911), and plasma biomarker levels (Aβ_42/40_, GFAP, NfL, and pTau‐181) in cognitively normal Baltimore Longitudinal Study of Aging (BLSA) participants. Analyses were stratified by APOEε4 status, cardiometabolic diseases, education, sex, race, and obesity.

**Results:**

Late‐life GDF15 abundance was associated with ACD risk in the full ARIC sample (HR=1.61 per log_2_ increase; [95% CI: 1.36‐1.90]) and in all but one subgroup – obese individuals (Figure 1A‐B). GDF15 measured during late‐life predicted 7‐year ACD risk with an AUC of 0.63 (AUC for GDF15+Age: 0.71). Midlife GDF15 was also associated with ACD risk in the full ARIC sample (HR=1.55 per log_2_ increase; [1.32‐1.82]) and in each subgroup (Figure 1C‐D). GDF15 measured during midlife predicted 20‐year ACD risk with an AUC of 0.65 (AUC for GDF15+Age: 0.75). We replicated GDF15’s relationship with ACD risk in the UKB sample and found that GDF15 has a much stronger association with VaD (HR=1.76 per log_2_ increase; [1.48‐2.10]) compared to AD (HR=1.11; [1.01‐1.23]; Figure 1E). In the BLSA, higher GDF15 was significantly associated with lower total and regional brain volume, a pattern of accelerated structural brain aging (SPARE‐BA), and elevated plasma NfL and pTau181 (Figure 2A‐G).

**Conclusions:**

Plasma GDF15 is associated with incident dementia risk across multiple cohorts, particularly vascular dementia, independent of cardiometabolic diseases and obesity. While our analyses indicate that GDF15 may play a role in dementia risk as early as midlife, GDF15, alone, provided only modest accuracy for dementia prediction.